# Crystal structure and redox potentials of the tppz-bridged {RuCl(bpy)}^+^ dimer

**DOI:** 10.1107/S2056989018011258

**Published:** 2018-08-16

**Authors:** Francisca N. Rein, Weizhong Chen, Brian L. Scott, Reginaldo C. Rocha

**Affiliations:** a Los Alamos National Laboratory, Los Alamos, NM 87545, USA

**Keywords:** crystal structure, dinuclear complex, electrochemistry, tetra­(pyrid­yl)pyrazine, ruthenium precatalyst

## Abstract

We report the structural and electrochemical characterization of the binuclear complex [μ-(C_24_H_16_N_6_){RuCl(C_10_H_8_N_2_)}_2_](PF_6_)_2_, which contains the bis-tridentate bridging ligand 2,3,5,6-tetra­kis­(pyridin-2-yl)pyrazine (tppz), the monodentate ligand Cl^−^, and the bidentate ligand 2,2′-bi­pyridine (bpy) {systematic name: μ-2,3,5,6-tetra­kis­(pyridin-2-yl)pyrazine-bis­[(2,2′-bi­pyridine)­chlorido­ru­thenium(II)] bis­(hexa­fluorido­phosphate)}. The complete [(bpy)(Cl)Ru(tppz)Ru(Cl)(bpy)]^2+^ dication is generated by crystallographic twofold symmetry; the tppz bridging ligand has a significantly twisted conformation, with an average angle of 42.4° between the mean planes of adjacent pyridyl rings. The metal-coordinated chloride ligands are in a *trans* configuration relative to each other across the {Ru(tppz)Ru} unit. The Ru^II^ ion exhibits a distorted octa­hedral geometry due to the restricted bite angle [160.6 (3)°] of the tppz ligand. For bpy, the bond lengths of the Ru—N bonds are 2.053 (8) and 2.090 (8) Å, with the shorter bond being opposite to Ru—Cl. For the tridentate tppz, the Ru—N distances involving the outer N atoms *trans* to each other are 2.069 (8) and 2.072 (9) Å, whereas the Ru—N bond involving the central N atom has the much shorter length of 1.939 (7) Å as a result of the geometric constraints and stronger π-acceptor ability of the pyrazine-centered bridge. The Ru—Cl distance is 2.407 (3) Å and the intra­molecular distance between Ru centers is 6.579 (4) Å. In the crystal, weak C—H⋯Cl and C—H⋯F inter­actions consolidate the packing.

## Abstract 2

The dinuclear complex [(bpy)(Cl)Ru(tppz)Ru(Cl)(bpy)](PF_6_)_2_ has been synthesized as a catalyst precursor and characterized by X-ray crystallography and cyclic voltammetry.

## Chemical context   

The design and synthesis of electrochemically and photochemically active ruthenium(II)–polypyridine complexes have been of continued inter­est in the development of homogeneous electrocatalysis and photocatalysis toward water-splitting schemes for renewable energy applications (Yamazaki *et al.*, 2010 ▸; Herrero *et al.*, 2011 ▸; Jurss *et al.*, 2012 ▸). In our previous work, we introduced Ru dyads in which a light-harvesting Ru moiety (chromophore) and a multi-electron/multi-proton redox-active Ru moiety (catalyst) were linked by back-to-back terpyridine (tpy–tpy) or tetra­pyrid­ylpyrazine (tppz) ligands to give modular light-driven oxidation catalysts with a varying extent of charge delocalization between the Ru centers (Chen *et al.*, 2009 ▸, 2013 ▸). In such catalysts containing the {(tpy/tppz)Ru(bpy)(*L*)} moiety (*L* = H_2_O or Cl^−^), the aqua species is typically formed by ligand substitution from its chloro precursor in water (Davidson *et al.*, 2015*b* ▸; Matias *et al.*, 2016 ▸). Therefore, the chloro complex reported here was initially prepared and isolated as an inter­mediate in the synthesis of binuclear precatalysts based on the {Ru(tppz)Ru} structural framework (Chen *et al.*, 2011 ▸). In addition to catalysis, the bis-tridentate tppz ligand finds relevance to the assembly of donor–acceptor metal complexes with electron/energy-transfer properties for potential applications in mol­ecular (opto)electronic devices (Davidson *et al.*, 2015*a* ▸; Fantacci *et al.*, 2004 ▸; Nagashima *et al.*, 2014 ▸, 2016 ▸; Wadman *et al.*, 2009 ▸).
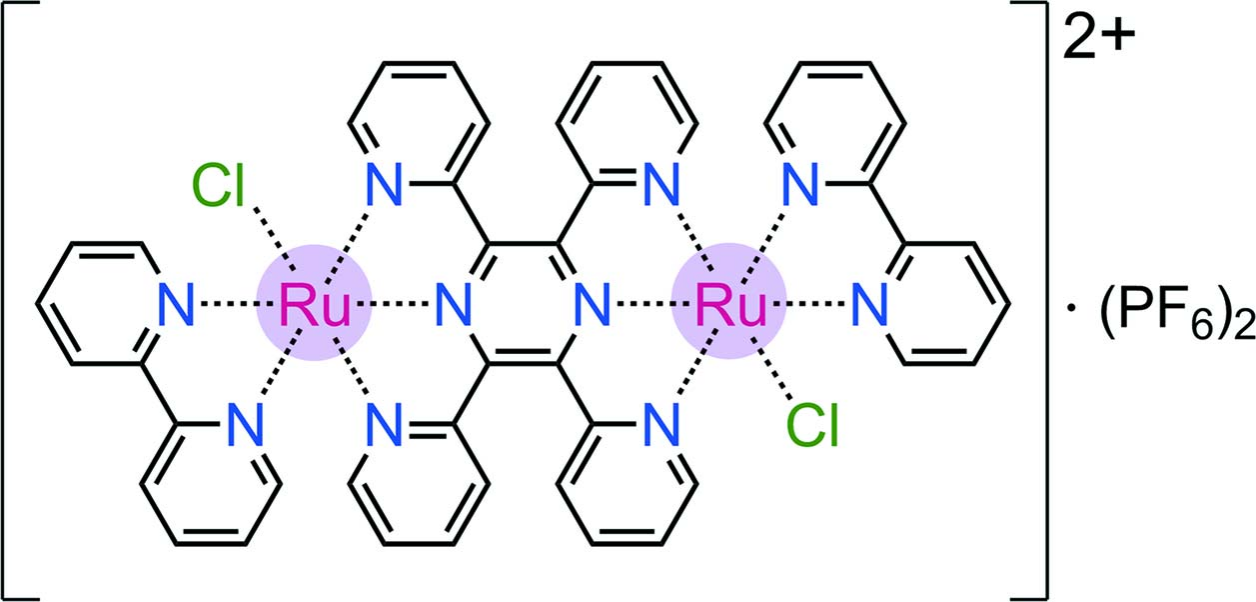


## Structural commentary   

**Figure 1:**
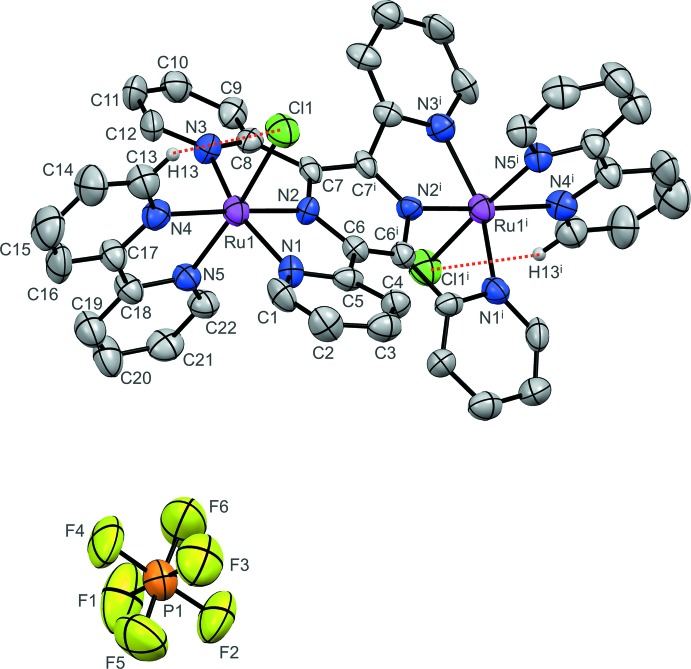
The mol­ecular structure of the title compound, with displacement ellipsoids drawn at the 40% probability level. H atoms have been omitted for clarity, except for H13; its close contact with Cl1 is indicated by a red dotted line. [Symmetry code: (i) −*x* + 1, *y*, −*z* + 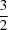.]

**Figure 2:**
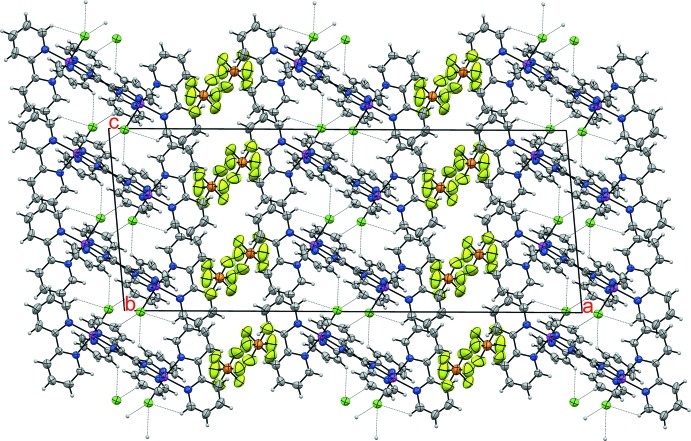
View along the *b* axis of a 1 × 2 × 2 crystal packing diagram of **I**. Displacement ellipsoids are drawn at the 40% probability level. Intra- and inter­molecular H⋯Cl inter­actions (those with separations shorter than the sum of van der Waals radii) are represented by the fine dotted lines.

The hexa­fluorido­phosphate salt of the binuclear complex [(bpy)(Cl)Ru^II^(μ-tppz)Ru^II^(Cl)(bpy)]^2+^ (**I**) crystallized from an aceto­nitrile solution in the monoclinic (*C*2/*c*) space group. Its crystal structure is shown in Fig. 1 ▸, and selected geometrical data are summarized in Table 1 ▸. As shown in Fig. 2 ▸, the dicationic complex packs in alternating layers with the uncoordinated PF_6_^−^ anions. The complete complex is generated by a crystallographic twofold axis bis­ecting the C6—C6^i^ and C7—C7^i^ [symmetry code: (i) −*x* + 1, *y*, −*z* + 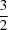] bonds of the central pyrazine ring, although it is close to being locally centrosymmetric. The complete tppz ligand has a significantly twisted conformation, with an average angle of 42.4° between the mean planes of adjacent pyridyl rings. The metal-coordinated chloride ligands are in a *trans* configuration relative to each other across the {Ru(tppz)Ru} core. The two equivalent metal coordination spheres exhibit a distorted octa­hedral geometry at the Ru^II^ ion due to the restricted bite angle of the bis-tridendate tppz ligand; the N1—Ru—N3 angle of 160.6 (3)° is very similar to those of related tppz–Ru^II^ complexes (Chen *et al.*, 2011 ▸; Jude *et al.*, 2013 ▸), and significantly less than the ideal angle of 180°. The Ru atom is essentially in the equatorial mean plane formed by atoms N1, N2, N3, and N4, with a deviation of only 0.026 Å. The bidentate bpy ligand has a *cis* configuration, with the N4—Ru—N5 angle of 78.4 (3)°, in agreement with those found in similar chlorido Ru^II^–bpy complexes (Chen *et al.*, 2013 ▸; Rein *et al.*, 2015 ▸). The N5 atom of bpy is arranged *trans* to the chloride ligand in a nearly linear N—Ru—Cl fashion [172.6 (2)°]. The distances of the two Ru—N bonds for bpy are 2.053 (8) and 2.090 (8) Å, with the shorter bond opposite to Ru—Cl reflecting the increased Ru^II^→N_bpy_ π-backbonding inter­action at the coordinating atom *trans* to the π-donor Cl^−^ ligand (Chen *et al.*, 2013 ▸). The Ru—Cl bond length of 2.406 (3) Å and the intra­molecular Ru⋯Ru separation of 6.579 (4) Å are also similar to those observed for the most closely related Ru(tppz)Ru complexes (Chen *et al.*, 2011 ▸; Hartshorn *et al.*, 1999 ▸). For the tridentate tppz ligand, the Ru—N bond lengths involving the outer N atoms *trans* to each other are 2.069 (8) and 2.070 (9) Å, whereas the Ru—N bond involving the central N atom has the much shorter length of 1.939 (7) Å as a result of both the geometric constraint imposed by such *mer*-arranged ligands and the stronger π-acceptor ability of the pyrazine-centered bridge (Chen *et al.*, 2011 ▸; Jude *et al.*, 2013 ▸). An intra­molecular C13—H13⋯Cl1 close contact of 2.74 Å is similar to that observed earlier for complexes containing the {RuCl(bpy)} moiety (Chen *et al.*, 2013 ▸; Jude *et al.*, 2008 ▸; Rein *et al.*, 2015 ▸), although this proximity appears to be partly a consequence of geometry rather than chemically significant bonding.

## Supra­molecular features   

In the crystal, C—H⋯Cl and C—H⋯F inter­actions (Table 2 ▸) with H⋯*X* distances that are shorter than the sum of van der Waals radii can be identified and appear to provide some further stabilization of the crystal packing.

## Database survey   

A search in the Cambridge Structural Database (Groom *et al.*, 2016 ▸) listed only four entries for the {RuCl(bpy)(tppz)} substructure. Of these, two are mononuclear complexes [one with the Ru^III^ oxidation state (Daryanavard *et al.*, 2009 ▸) and another at the Ru^II^ state (Tondreau *et al.*, 1996 ▸)] and the other two are binuclear complexes [one with tpy instead of bpy and Cl^−^ (Chen *et al.*, 2011 ▸), and another with Me_2_bpy instead of bpy and the two Cl^−^ ligands in a *cis* configuration (Hartshorn *et al.*, 1999 ▸)].

## Electrochemical characterization   

**Figure 3:**
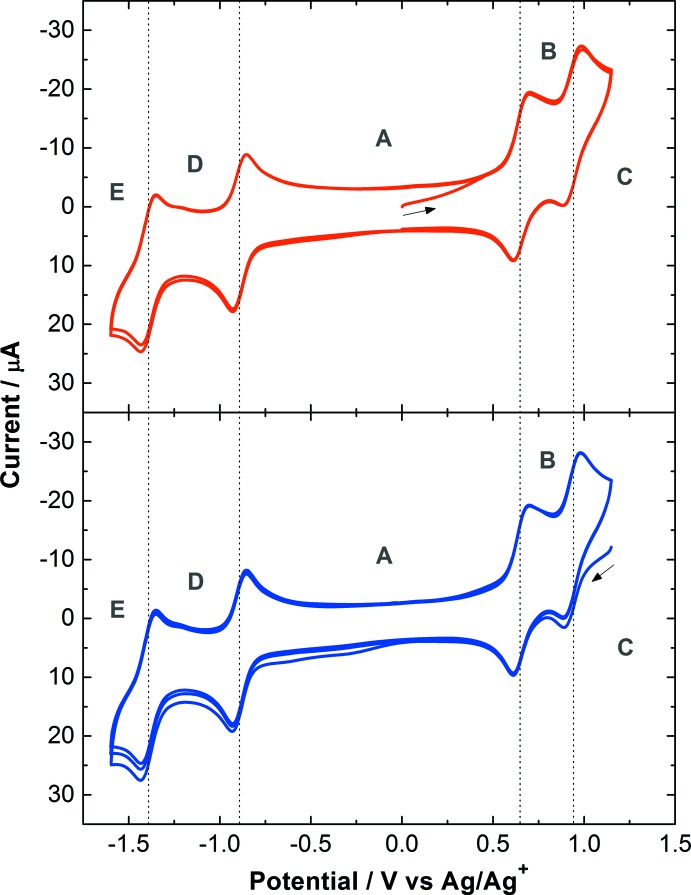
Cyclic voltammograms of 1.0 m*M* solutions of **I** in aceto­nitrile with 0.1 *M* Bu_4_NPF_6_ as electrolyte. The various redox states are represented by the potential regions as **A**: [(bpy)(Cl)Ru^II^(tppz)Ru^II^(Cl)(bpy)]^2+^, **B**: [(bpy)(Cl)Ru^II^(tppz)Ru^III^(Cl)(bpy)]^3+^, **C**: [(bpy)(Cl)Ru^III^(tppz)Ru^III^(Cl)(bpy)]^4+^, **D**: [(bpy)(Cl)Ru^II^(tppz^−^)Ru^II^(Cl)(bpy)]^+^, and **E**: [(bpy)(Cl)Ru^II^(tppz^2−^)Ru^II^(Cl)(bpy)].

Cyclic voltammograms of **I** in aceto­nitrile (Fig. 3 ▸; top) show two metal-based oxidation processes at +0.65 and +0.94 V *versus* Ag/Ag^+^ (10 m*M* AgNO_3_). These processes are clearly reversible and correspond to the redox couples Ru^II^–Ru^II^/Ru^II^–Ru^III^ and Ru^II^–Ru^III^/Ru^III^–Ru^III^, respectively. The stability of the fully oxidized complex is also demonstrated by the voltammogram starting from the Ru^III^–Ru^III^ species, obtained after application of +1.25 V for 100 s prior to the initial run in the cathodic direction (Fig. 3 ▸; bottom). Two additional reversible processes are observed at −0.89 and −1.39 V, which are characteristic of the ligand-based reductions at the tppz bridge. The separation of 290 mV between the two Ru^II^/Ru^III^ redox potentials gives a comproportionation constant (*K*_c_) of about 8.0 × 10^4^, which reflects the stabilization of the mixed-valent state Ru^II^–Ru^III^ relative to its reduced and oxidized isovalent counterparts Ru^II^–Ru^II^ and Ru^III^–Ru^III^ (Richardson & Taube, 1984 ▸; Rocha & Toma, 2004 ▸). This *K*_c_ value suggests a significant communication between the Ru centers, although electrochemical properties alone cannot be taken as conclusive evidence for electronic coupling across the bridging ligand because of possible electrostatic effects (Jude *et al.*, 2008 ▸). By comparison with its precursor [Cl_3_Ru^II^(tppz)Ru^III^Cl_3_]^−^, which shows a separation greater than 700 mV between the two Ru^II^/Ru^III^ redox potentials and which has been well characterized as a borderline case of valence localization/delocalization (Concepcion *et al.*, 2008 ▸; Rocha *et al.*, 2008 ▸), the electrochemical data are consistent with a charge-localized configuration in the mixed-valent species [(bpy)(Cl)Ru^II^(tppz)Ru^III^(Cl)(bpy)]^3+^.

## Synthesis and crystallization   

Compound **I** was prepared from the mixed-valent complex (*n*Bu_4_N)[Cl_3_Ru^II^(tppz)Ru^III^Cl_3_] as starting material (Rocha *et al.*, 2008 ▸). This precursor was treated by refluxing an ethano­lic solution with two equivalents of bpy in the presence of tri­ethyl­amine as a reductant and the final solid product was collected by filtration of the precipitate formed upon addition of a concentrated aqueous solution of NH_4_PF_6_ to the reaction mixture. Green blocks of **I** were grown by the slow diffusion of diethyl ether into aceto­nitrile solutions of the product in long thin tubes.

## Refinement   

Crystal data, data collection, and structure refinement details are summarized in Table 3 ▸. Six disordered aceto­nitrile solvent mol­ecules were treated using *PLATON*/SQUEEZE (van der Sluis & Spek, 1990 ▸; Spek, 2015 ▸) and not included in the refinement model; the stated chemical formula, molar mass, *etc*., do not take account of these solvent mol­ecules. All H atoms (aromatic) were idealized and refined as riding atoms, with C—H = 0.93 Å and *U*_iso_(H) = 1.2*U*_eq_(C).

## Supplementary Material

Crystal structure: contains datablock(s) I. DOI: 10.1107/S2056989018011258/hb7761sup1.cif

Structure factors: contains datablock(s) I. DOI: 10.1107/S2056989018011258/hb7761Isup2.hkl

CCDC reference: 1860573

Additional supporting information:  crystallographic information; 3D view; checkCIF report

## Figures and Tables

**Table 1:** Selected geometric parameters (Å, °)

Ru1—N2	1.939 (7)	Ru1—N1	2.070 (9)
Ru1—N5	2.053 (8)	Ru1—N4	2.090 (8)
Ru1—N3	2.069 (8)	Ru1—Cl1	2.406 (3)
			
N2—Ru1—N5	98.1 (3)	N3—Ru1—N4	99.3 (3)
N2—Ru1—N3	80.0 (3)	N1—Ru1—N4	100.1 (3)
N5—Ru1—N3	88.5 (3)	N2—Ru1—Cl1	89.2 (2)
N2—Ru1—N1	80.6 (3)	N5—Ru1—Cl1	172.6 (2)
N5—Ru1—N1	95.4 (3)	N3—Ru1—Cl1	91.7 (2)
N3—Ru1—N1	160.6 (3)	N1—Ru1—Cl1	86.8 (2)
N2—Ru1—N4	176.4 (3)	N4—Ru1—Cl1	94.3 (3)
N5—Ru1—N4	78.4 (3)		

**Table 2:** Hydrogen-bond geometry (Å, °)

*D*—H⋯*A*	*D*—H	H⋯*A*	*D*⋯*A*	*D*—H⋯*A*
C13—H13⋯Cl1	0.93	2.74	3.362 (12)	125
C9—H9⋯Cl1^i^	0.93	2.71	3.390 (11)	131
C14—H14⋯F2^ii^	0.93	2.54	3.342 (17)	144

Symmetry codes: (i) 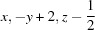; (ii) 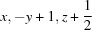.

**Table 3:** Experimental details

Crystal data	
Chemical formula	[Ru_2_Cl_2_(C_10_H_8_N_2_)_2_(C_24_H_16_N_6_)](PF_6_)_2_
*M*_r_	1311.77
Crystal system, space group	Monoclinic, *C*2/*c*
Temperature (K)	100
*a*, *b*, *c* (Å)	36.04 (3), 11.517 (11), 14.406 (14)
β (°)	95.258 (13)
*V* (Å^3^)	5954 (10)
*Z*	4
Radiation type	Mo *K*α
μ (mm^−1^)	0.73
Crystal size (mm)	0.20 × 0.14 × 0.06

Data collection	
Diffractometer	Bruker D8 with APEXII CCD
Absorption correction	Multi-scan (*SADABS*; Sheldrick, 2008 ▸)
*T*_min_, *T*_max_	0.862, 0.956
No. of measured, independent and observed [*I* > 2σ(*I*)] reflections	13828, 5306, 2167
*R*_int_	0.121
(sin θ/λ)_max_ (Å^−1^)	0.600

Refinement	
*R*[*F* ^2^ > 2σ(*F* ^2^)], *wR*(*F* ^2^), *S*	0.078, 0.221, 0.92
No. of reflections	5306
No. of parameters	325
H-atom treatment	H-atom parameters constrained
Δρ_max_, Δρ_min_ (e Å^−3^)	0.89, −0.52

Computer programs: *APEXII* and *SAINT-Plus* (Bruker, 2009 ▸), *SHELXS2013* (Sheldrick, 2015*a* ▸), *SHELXL2014* (Sheldrick, 2015*b* ▸), *Mercury* (Macrae *et al.*, 2008 ▸) and *publCIF* (Westrip, 2010 ▸).

## supplementary crystallographic information

### Crystal data

**Table:**

[Ru_2_Cl_2_(C_10_H_8_N_2_)_2_(C_24_H_16_N_6_)](PF_6_)_2_	*F*(000) = 2600
*M**_r_* = 1311.77	*D*_x_ = 1.463 Mg m^−^^3^
Monoclinic, *C*2/*c*	Mo *K*α radiation, λ = 0.71073 Å
*a* = 36.04 (3) Å	Cell parameters from 792 reflections
*b* = 11.517 (11) Å	θ = 2.3–15.6°
*c* = 14.406 (14) Å	µ = 0.73 mm^−^^1^
β = 95.258 (13)°	*T* = 100 K
*V* = 5954 (10) Å^3^	Block, green
*Z* = 4	0.20 × 0.14 × 0.06 mm

### Data collection

**Table:**

Bruker D8 with APEXII CCD diffractometer	2167 reflections with *I* > 2σ(*I*)
ω scans	*R*_int_ = 0.121
Absorption correction: multi-scan (SADABS; Sheldrick, 2008)	θ_max_ = 25.3°, θ_min_ = 1.9°
*T*_min_ = 0.862, *T*_max_ = 0.956	*h* = −43→41
13828 measured reflections	*k* = −13→12
5306 independent reflections	*l* = −17→9

### Refinement

**Table:**

Refinement on *F*^2^	Primary atom site location: structure-invariant direct methods
Least-squares matrix: full	Hydrogen site location: inferred from neighbouring sites
*R*[*F*^2^ > 2σ(*F*^2^)] = 0.078	H-atom parameters constrained
*wR*(*F*^2^) = 0.221	*w* = 1/[σ^2^(*F*_o_^2^) + (0.1018*P*)^2^] where *P* = (*F*_o_^2^ + 2*F*_c_^2^)/3
*S* = 0.92	(Δ/σ)_max_ = 0.001
5306 reflections	Δρ_max_ = 0.89 e Å^−^^3^
325 parameters	Δρ_min_ = −0.52 e Å^−^^3^
0 restraints	

### Special details

**Table:**

Geometry. All esds (except the esd in the dihedral angle between two l.s. planes) are estimated using the full covariance matrix. The cell esds are taken into account individually in the estimation of esds in distances, angles and torsion angles; correlations between esds in cell parameters are only used when they are defined by crystal symmetry. An approximate (isotropic) treatment of cell esds is used for estimating esds involving l.s. planes.

### Fractional atomic coordinates and isotropic or equivalent isotropic displacement parameters (Å^2^)

**Table:**

	*x*	*y*	*z*	*U*_iso_*/*U*_eq_	
Ru1	0.42533 (2)	0.80820 (7)	0.86533 (6)	0.0515 (3)	
P1	0.28591 (11)	0.1287 (4)	0.8171 (3)	0.0899 (11)	
Cl1	0.46532 (8)	0.8343 (3)	1.00695 (19)	0.0738 (9)	
N1	0.4391 (2)	0.6338 (7)	0.8747 (6)	0.052 (2)	
N2	0.46892 (19)	0.8090 (7)	0.7958 (5)	0.0411 (18)	
N3	0.4279 (2)	0.9831 (7)	0.8337 (5)	0.054 (2)	
N4	0.3763 (2)	0.8077 (7)	0.9324 (6)	0.056 (2)	
N5	0.3856 (2)	0.7895 (6)	0.7548 (6)	0.050 (2)	
F1	0.2644 (3)	0.1831 (10)	0.7306 (6)	0.192 (5)	
F2	0.3108 (2)	0.0590 (8)	0.7523 (6)	0.145 (3)	
F3	0.3087 (3)	0.0751 (8)	0.9044 (6)	0.156 (3)	
F4	0.2625 (2)	0.1970 (8)	0.8839 (6)	0.146 (3)	
F5	0.2599 (3)	0.0239 (10)	0.8153 (7)	0.182 (4)	
F6	0.3144 (3)	0.2278 (8)	0.8224 (8)	0.178 (4)	
C1	0.4250 (3)	0.5509 (11)	0.9278 (7)	0.063 (3)	
H1	0.403164	0.567077	0.954866	0.076*	
C2	0.4409 (3)	0.4466 (11)	0.9435 (8)	0.071 (3)	
H2	0.429611	0.390445	0.977902	0.085*	
C3	0.4743 (3)	0.4249 (9)	0.9073 (7)	0.064 (3)	
H3	0.486337	0.354441	0.919608	0.077*	
C4	0.4900 (3)	0.5057 (9)	0.8535 (7)	0.053 (3)	
H4	0.512875	0.492082	0.830629	0.064*	
C5	0.4710 (3)	0.6078 (8)	0.8339 (6)	0.047 (3)	
C6	0.4860 (2)	0.7048 (9)	0.7808 (6)	0.044 (2)	
C7	0.4829 (2)	0.9123 (9)	0.7690 (6)	0.043 (2)	
C8	0.4563 (3)	1.0102 (9)	0.7801 (6)	0.045 (2)	
C9	0.4566 (3)	1.1141 (10)	0.7356 (7)	0.061 (3)	
H9	0.474145	1.128530	0.693447	0.073*	
C10	0.4312 (3)	1.1972 (10)	0.7528 (8)	0.066 (3)	
H10	0.432617	1.270606	0.726425	0.079*	
C11	0.4039 (3)	1.1725 (11)	0.8086 (8)	0.071 (3)	
H11	0.386399	1.228549	0.820873	0.085*	
C12	0.4024 (3)	1.0628 (11)	0.8467 (7)	0.063 (3)	
H12	0.382958	1.044423	0.882405	0.076*	
C13	0.3731 (3)	0.8161 (10)	1.0246 (8)	0.072 (3)	
H13	0.395001	0.822036	1.063895	0.086*	
C14	0.3411 (4)	0.8166 (11)	1.0637 (10)	0.090 (4)	
H14	0.340712	0.826684	1.127676	0.108*	
C15	0.3084 (4)	0.8020 (12)	1.0072 (11)	0.101 (5)	
H15	0.285660	0.798244	1.032786	0.121*	
C16	0.3100 (3)	0.7929 (10)	0.9126 (10)	0.082 (4)	
H16	0.288200	0.783580	0.873405	0.099*	
C17	0.3444 (3)	0.7977 (9)	0.8752 (7)	0.057 (3)	
C18	0.3493 (3)	0.7923 (9)	0.7757 (7)	0.058 (3)	
C19	0.3204 (3)	0.7896 (10)	0.7048 (9)	0.078 (4)	
H19	0.295778	0.789111	0.719650	0.093*	
C20	0.3277 (3)	0.7878 (11)	0.6156 (10)	0.086 (4)	
H20	0.308295	0.787549	0.568362	0.103*	
C21	0.3638 (3)	0.7864 (9)	0.5938 (7)	0.072 (3)	
H21	0.369245	0.783504	0.531922	0.086*	
C22	0.3924 (3)	0.7893 (8)	0.6657 (7)	0.059 (3)	
H22	0.416987	0.791088	0.650899	0.070*	

### Atomic displacement parameters (Å^2^)

**Table:**

	*U*^11^	*U*^22^	*U*^33^	*U*^12^	*U*^13^	*U*^23^
Ru1	0.0439 (5)	0.0600 (6)	0.0513 (6)	0.0015 (5)	0.0081 (4)	−0.0001 (5)
P1	0.074 (2)	0.104 (3)	0.093 (3)	0.005 (3)	0.011 (2)	0.006 (2)
Cl1	0.0681 (18)	0.091 (2)	0.0614 (19)	−0.0025 (17)	0.0022 (14)	−0.0059 (16)
N1	0.042 (5)	0.063 (6)	0.052 (6)	0.009 (5)	0.007 (4)	0.006 (5)
N2	0.041 (4)	0.038 (5)	0.045 (5)	0.008 (4)	0.009 (4)	0.003 (4)
N3	0.053 (5)	0.052 (6)	0.057 (6)	0.007 (5)	0.011 (4)	−0.001 (4)
N4	0.060 (5)	0.061 (6)	0.049 (6)	0.007 (5)	0.007 (5)	−0.002 (5)
N5	0.050 (5)	0.057 (6)	0.043 (5)	−0.002 (4)	0.005 (4)	−0.003 (4)
F1	0.197 (10)	0.263 (13)	0.117 (7)	0.127 (9)	0.014 (7)	0.058 (7)
F2	0.141 (7)	0.162 (8)	0.139 (7)	0.053 (6)	0.051 (6)	−0.020 (6)
F3	0.160 (8)	0.189 (9)	0.115 (7)	0.034 (7)	−0.005 (6)	0.006 (6)
F4	0.115 (6)	0.194 (10)	0.135 (7)	0.052 (6)	0.040 (6)	−0.038 (6)
F5	0.133 (8)	0.219 (11)	0.199 (10)	−0.073 (8)	0.035 (7)	−0.039 (8)
F6	0.131 (8)	0.122 (8)	0.288 (13)	−0.019 (7)	0.055 (8)	−0.008 (8)
C1	0.038 (6)	0.087 (9)	0.065 (8)	−0.015 (7)	0.009 (5)	0.006 (7)
C2	0.071 (9)	0.065 (9)	0.075 (9)	−0.006 (7)	0.002 (7)	0.021 (7)
C3	0.077 (8)	0.049 (7)	0.064 (8)	−0.001 (7)	−0.005 (6)	0.003 (6)
C4	0.054 (6)	0.048 (7)	0.058 (7)	−0.002 (6)	0.005 (5)	0.004 (6)
C5	0.057 (7)	0.036 (6)	0.046 (7)	−0.005 (5)	−0.010 (5)	0.002 (5)
C6	0.037 (5)	0.050 (7)	0.045 (6)	−0.004 (5)	−0.001 (4)	0.000 (5)
C7	0.030 (5)	0.062 (7)	0.035 (6)	−0.001 (5)	−0.006 (4)	−0.001 (5)
C8	0.048 (6)	0.048 (7)	0.037 (6)	0.005 (5)	−0.001 (5)	−0.004 (5)
C9	0.064 (7)	0.070 (8)	0.046 (7)	0.014 (7)	−0.006 (5)	0.001 (6)
C10	0.063 (7)	0.060 (8)	0.072 (8)	0.005 (7)	−0.011 (6)	0.003 (6)
C11	0.051 (7)	0.062 (9)	0.099 (10)	0.020 (7)	−0.001 (7)	−0.002 (7)
C12	0.036 (6)	0.080 (9)	0.074 (8)	0.010 (6)	0.005 (5)	−0.016 (7)
C13	0.065 (8)	0.082 (9)	0.068 (8)	0.001 (7)	0.006 (7)	0.003 (7)
C14	0.068 (9)	0.112 (11)	0.095 (10)	−0.004 (9)	0.036 (8)	−0.004 (8)
C15	0.082 (10)	0.127 (13)	0.099 (12)	0.011 (10)	0.041 (9)	−0.006 (10)
C16	0.048 (7)	0.087 (10)	0.115 (12)	0.009 (7)	0.030 (7)	0.004 (8)
C17	0.046 (6)	0.078 (8)	0.048 (7)	0.000 (6)	0.002 (5)	−0.001 (6)
C18	0.049 (6)	0.073 (8)	0.051 (7)	−0.005 (6)	−0.004 (5)	0.001 (6)
C19	0.058 (7)	0.106 (11)	0.070 (9)	0.001 (7)	0.005 (7)	−0.003 (8)
C20	0.056 (8)	0.124 (12)	0.076 (10)	0.014 (8)	−0.009 (7)	0.008 (9)
C21	0.089 (9)	0.082 (9)	0.044 (7)	0.002 (7)	0.001 (7)	−0.002 (6)
C22	0.058 (7)	0.067 (8)	0.051 (7)	−0.004 (6)	0.004 (6)	−0.007 (6)

### Geometric parameters (Å, º)

**Table:**

Ru1—N2	1.939 (7)	C5—C6	1.484 (12)
Ru1—N5	2.053 (8)	C6—C6^i^	1.403 (17)
Ru1—N3	2.069 (8)	C7—C7^i^	1.396 (16)
Ru1—N1	2.070 (9)	C7—C8	1.497 (12)
Ru1—N4	2.090 (8)	C8—C9	1.358 (13)
Ru1—Cl1	2.406 (3)	C9—C10	1.363 (13)
P1—F5	1.527 (10)	C9—H9	0.9300
P1—F6	1.532 (10)	C10—C11	1.358 (14)
P1—F1	1.539 (9)	C10—H10	0.9300
P1—F4	1.552 (8)	C11—C12	1.380 (14)
P1—F3	1.565 (9)	C11—H11	0.9300
P1—F2	1.574 (8)	C12—H12	0.9300
N1—C1	1.351 (12)	C13—C14	1.331 (14)
N1—C5	1.370 (11)	C13—H13	0.9300
N2—C7	1.361 (10)	C14—C15	1.379 (17)
N2—C6	1.375 (10)	C14—H14	0.9300
N3—C12	1.326 (11)	C15—C16	1.374 (16)
N3—C8	1.374 (11)	C15—H15	0.9300
N4—C13	1.347 (12)	C16—C17	1.399 (14)
N4—C17	1.355 (12)	C16—H16	0.9300
N5—C22	1.330 (11)	C17—C18	1.462 (13)
N5—C18	1.367 (12)	C18—C19	1.392 (14)
C1—C2	1.340 (14)	C19—C20	1.337 (14)
C1—H1	0.9300	C19—H19	0.9300
C2—C3	1.380 (13)	C20—C21	1.367 (14)
C2—H2	0.9300	C20—H20	0.9300
C3—C4	1.365 (13)	C21—C22	1.392 (13)
C3—H3	0.9300	C21—H21	0.9300
C4—C5	1.376 (12)	C22—H22	0.9300
C4—H4	0.9300		
			
N2—Ru1—N5	98.1 (3)	C5—C4—H4	120.8
N2—Ru1—N3	80.0 (3)	N1—C5—C4	121.3 (9)
N5—Ru1—N3	88.5 (3)	N1—C5—C6	114.6 (8)
N2—Ru1—N1	80.6 (3)	C4—C5—C6	123.4 (10)
N5—Ru1—N1	95.4 (3)	N2—C6—C6^i^	117.2 (5)
N3—Ru1—N1	160.6 (3)	N2—C6—C5	112.7 (8)
N2—Ru1—N4	176.4 (3)	C6^i^—C6—C5	130.1 (6)
N5—Ru1—N4	78.4 (3)	N2—C7—C7^i^	118.3 (5)
N3—Ru1—N4	99.3 (3)	N2—C7—C8	111.9 (7)
N1—Ru1—N4	100.1 (3)	C7^i^—C7—C8	129.7 (6)
N2—Ru1—Cl1	89.2 (2)	C9—C8—N3	120.2 (9)
N5—Ru1—Cl1	172.6 (2)	C9—C8—C7	125.5 (10)
N3—Ru1—Cl1	91.7 (2)	N3—C8—C7	113.9 (8)
N1—Ru1—Cl1	86.8 (2)	C8—C9—C10	119.9 (11)
N4—Ru1—Cl1	94.3 (3)	C8—C9—H9	120.0
F5—P1—F6	175.7 (7)	C10—C9—H9	120.0
F5—P1—F1	92.6 (7)	C11—C10—C9	119.8 (11)
F6—P1—F1	91.2 (7)	C11—C10—H10	120.1
F5—P1—F4	92.5 (6)	C9—C10—H10	120.1
F6—P1—F4	89.5 (6)	C10—C11—C12	119.0 (11)
F1—P1—F4	91.9 (5)	C10—C11—H11	120.5
F5—P1—F3	88.8 (6)	C12—C11—H11	120.5
F6—P1—F3	87.4 (6)	N3—C12—C11	121.6 (10)
F1—P1—F3	178.4 (7)	N3—C12—H12	119.2
F4—P1—F3	88.7 (5)	C11—C12—H12	119.2
F5—P1—F2	88.3 (6)	C14—C13—N4	125.0 (11)
F6—P1—F2	89.6 (6)	C14—C13—H13	117.5
F1—P1—F2	89.9 (5)	N4—C13—H13	117.5
F4—P1—F2	178.0 (6)	C13—C14—C15	118.5 (13)
F3—P1—F2	89.4 (5)	C13—C14—H14	120.8
C1—N1—C5	117.4 (9)	C15—C14—H14	120.8
C1—N1—Ru1	128.5 (7)	C16—C15—C14	119.0 (12)
C5—N1—Ru1	112.9 (7)	C16—C15—H15	120.5
C7—N2—C6	122.4 (7)	C14—C15—H15	120.5
C7—N2—Ru1	119.2 (6)	C15—C16—C17	119.8 (12)
C6—N2—Ru1	118.2 (6)	C15—C16—H16	120.1
C12—N3—C8	119.0 (9)	C17—C16—H16	120.1
C12—N3—Ru1	126.7 (8)	N4—C17—C16	120.1 (10)
C8—N3—Ru1	113.3 (6)	N4—C17—C18	115.3 (9)
C13—N4—C17	117.5 (9)	C16—C17—C18	124.5 (10)
C13—N4—Ru1	127.4 (7)	N5—C18—C19	120.4 (10)
C17—N4—Ru1	115.1 (7)	N5—C18—C17	114.8 (8)
C22—N5—C18	118.5 (8)	C19—C18—C17	124.8 (10)
C22—N5—Ru1	124.8 (7)	C20—C19—C18	120.3 (11)
C18—N5—Ru1	116.1 (6)	C20—C19—H19	119.9
C2—C1—N1	123.6 (10)	C18—C19—H19	119.9
C2—C1—H1	118.2	C19—C20—C21	119.9 (11)
N1—C1—H1	118.2	C19—C20—H20	120.1
C1—C2—C3	118.1 (11)	C21—C20—H20	120.1
C1—C2—H2	120.9	C20—C21—C22	119.0 (11)
C3—C2—H2	120.9	C20—C21—H21	120.5
C4—C3—C2	120.8 (11)	C22—C21—H21	120.5
C4—C3—H3	119.6	N5—C22—C21	121.9 (10)
C2—C3—H3	119.6	N5—C22—H22	119.1
C3—C4—C5	118.3 (10)	C21—C22—H22	119.1
C3—C4—H4	120.8		
			
C5—N1—C1—C2	1.1 (15)	C7—C8—C9—C10	−179.0 (9)
Ru1—N1—C1—C2	−165.8 (8)	C8—C9—C10—C11	−5.3 (15)
N1—C1—C2—C3	3.3 (16)	C9—C10—C11—C12	−0.1 (16)
C1—C2—C3—C4	−2.8 (16)	C8—N3—C12—C11	−0.7 (14)
C2—C3—C4—C5	−2.0 (15)	Ru1—N3—C12—C11	−168.5 (8)
C1—N1—C5—C4	−6.2 (13)	C10—C11—C12—N3	3.2 (16)
Ru1—N1—C5—C4	162.7 (7)	C17—N4—C13—C14	−0.7 (17)
C1—N1—C5—C6	−176.9 (8)	Ru1—N4—C13—C14	179.2 (10)
Ru1—N1—C5—C6	−8.0 (9)	N4—C13—C14—C15	3 (2)
C3—C4—C5—N1	6.6 (14)	C13—C14—C15—C16	−3 (2)
C3—C4—C5—C6	176.5 (9)	C14—C15—C16—C17	0.5 (19)
C7—N2—C6—C6^i^	−13.6 (14)	C13—N4—C17—C16	−2.0 (15)
Ru1—N2—C6—C6^i^	170.9 (8)	Ru1—N4—C17—C16	178.1 (8)
C7—N2—C6—C5	167.0 (7)	C13—N4—C17—C18	178.2 (9)
Ru1—N2—C6—C5	−8.6 (9)	Ru1—N4—C17—C18	−1.7 (11)
N1—C5—C6—N2	10.7 (11)	C15—C16—C17—N4	2.0 (17)
C4—C5—C6—N2	−159.7 (8)	C15—C16—C17—C18	−178.2 (11)
N1—C5—C6—C6^i^	−168.6 (11)	C22—N5—C18—C19	2.9 (14)
C4—C5—C6—C6^i^	20.9 (17)	Ru1—N5—C18—C19	174.9 (8)
C6—N2—C7—C7^i^	−4.5 (14)	C22—N5—C18—C17	−177.3 (8)
Ru1—N2—C7—C7^i^	171.0 (8)	Ru1—N5—C18—C17	−5.4 (11)
C6—N2—C7—C8	171.4 (7)	N4—C17—C18—N5	4.6 (13)
Ru1—N2—C7—C8	−13.0 (9)	C16—C17—C18—N5	−175.2 (10)
C12—N3—C8—C9	−4.8 (13)	N4—C17—C18—C19	−175.6 (10)
Ru1—N3—C8—C9	164.6 (7)	C16—C17—C18—C19	4.5 (18)
C12—N3—C8—C7	−178.7 (8)	N5—C18—C19—C20	−2.0 (17)
Ru1—N3—C8—C7	−9.4 (9)	C17—C18—C19—C20	178.2 (11)
N2—C7—C8—C9	−159.3 (9)	C18—C19—C20—C21	1.2 (19)
C7^i^—C7—C8—C9	16.1 (17)	C19—C20—C21—C22	−1.3 (18)
N2—C7—C8—N3	14.3 (10)	C18—N5—C22—C21	−3.0 (14)
C7^i^—C7—C8—N3	−170.3 (11)	Ru1—N5—C22—C21	−174.3 (7)
N3—C8—C9—C10	7.8 (14)	C20—C21—C22—N5	2.3 (16)

Symmetry code: (i) −*x*+1, *y*, −*z*+3/2.

### Hydrogen-bond geometry (Å, º)

**Table:**

*D*—H···*A*	*D*—H	H···*A*	*D*···*A*	*D*—H···*A*
C13—H13···Cl1	0.93	2.74	3.362 (12)	125
C9—H9···Cl1^ii^	0.93	2.71	3.390 (11)	131
C14—H14···F2^iii^	0.93	2.54	3.342 (17)	144

Symmetry codes: (ii) *x*, −*y*+2, *z*−1/2; (iii) *x*, −*y*+1, *z*+1/2.
